# Delayed Tooth Development and the Impaired Differentiation of Stem/Progenitor Cells in Incisors from Type 2 Diabetes Mice

**DOI:** 10.3390/ijms252413619

**Published:** 2024-12-19

**Authors:** Yoshifumi Kobayashi, Jia Huang, Brandon K. Barnett, Carla Y. Falcon, Paul A. Falcon, Craig S. Hirschberg, Daniel H. Fine, Yi Ye, Emi Shimizu

**Affiliations:** 1 Department of Oral Biology, Rutgers School of Dental Medicine, Newark, NJ 07103, USA; kobayayo@rwjms.rutgers.edu (Y.K.); jh1707@rutgers.edu (J.H.); finedh@sdm.rutgers.edu (D.H.F.); 2 Department of Endodontics, Rutgers School of Dental Medicine, Newark, NJ 07103, USAfalconcy@sdm.rutgers.edu (C.Y.F.); falconpa@sdm.rutgers.edu (P.A.F.); hirschcs@sdm.rutgers.edu (C.S.H.); 3 Bluestone Center for Clinical Research, New York University College of Dentistry, New York, NY 10010, USA; yy22@nyu.edu; 4 Department of Oral and Maxillofacial Surgery, New York University College of Dentistry, New York, NY 10010, USA; 5 Department of Molecular Pathobiology, New York University College of Dentistry, New York, NY 10010, USA

**Keywords:** type 2 diabetes, tooth development, dentin, enamel, dental pulp stem cells, odontoblast, ameloblast, transcriptome, RNA-Seq

## Abstract

Patients with diabetes mellitus (DM) have an increased risk of tooth decay caused by alterations in their tooth development and their oral environment, as well as a tendency to present with pulp infection due to compromised immune response. The present study analyzed the characteristic alterations in tooth development under DM conditions using incisors from *db/db* type 2 diabetic mouse model (T2DM mice). In micro-CT analyses, T2DM mice showed delayed dentin and enamel formation. Through transcriptomic analyses, pre-ameloblast- and pre-odontoblast-specific genes were found to be significantly decreased in the incisors of T2DM mice, whereas major ameloblast- and mature odontoblast-specific genes were not changed. Stem cell markers were decreased in T2DM mice compared to those from the control mice, suggesting that the stemness of dental pulp cells (DPCs) is attenuated in T2DM mice. In vitro analyses demonstrated that DPCs from T2DM mice have lower colony-forming units (CFU), slower propagation, and diminished differentiation characteristics compared to the control. These data suggest that T2DM conditions could impair the differentiation property of multiple progenitor/stem cells in the tooth, resulting in delayed tooth development in T2DM mice.

## 1. Introduction

Diabetes mellitus (DM) is a common group of metabolic disorders characterized by hyperglycemia due to the absolute or relative deficiencies of insulin action. Currently, DM is a global public health problem, with an estimated 463 million people around the world developing DM in 2019, and this number is projected to increase to 700 million by 2045 [1]. The Centers for Disease Control and Prevention (CDC) announced that 34.2 million individuals (10.5% of the US population) had diabetes in 2020. The most common type of diabetes mellitus is type 2 diabetes (T2DM), accounting for 90% to 95% of total DM patients. Obesity-induced T2DM is characterized primarily by chronic hyperglycemia and insulin resistance in peripheral tissues.

The conditions of DM negatively affect various aspects of oral health (reviewed in [2]). The incidence of periodontitis increases in DM patients due to the immune response attenuation caused by the accumulation of advanced glycosylation end products (AGE) and poor regenerative capacity. Maternal diabetes negatively impacts the enamel formation of offspring [3,4]. Also, xerostomia or hyposialia accompanied by DM makes the oral environment vulnerable to caries formation or infectious diseases such as candidiasis [5,6,7]. Furthermore, dental pulps from DM patients tend to present with limited dental collateral circulation, impaired immune response, and an increased risk of acquiring pulpal infection [8]. Several animal studies with type 1 diabetes models show that hyperglycemia inhibits tertiary dentin formation and ameloblast differentiation [6,9,10,11].

The influence of T2DM on developing teeth is evident in fetuses of diabetic pregnant women, as these mothers are primarily obligated to avoid medications for diabetes during pregnancy to prevent side effects to fetuses [12], resulting in an untreated diabetic condition. Diabetes patients during pregnancy consist of two groups: gestational diabetes mellitus (GDM) and pregnancy diabetes mellitus (PDM), with prevalences of approximately 7.8% in 2020 [13] and 1.1% in 2021 [14], respectively, in the US. In a prospective cohort study assessing 50 children of women with GDM and 250 children of normoglycemic women in Brazil, children of GDM mothers showed a 2.6-fold higher prevalence of developmental defects of enamel compared to the control group [3]. Further, a systematic review in 2022 clarified that children of mothers with GDM have enamel hypo-mineralization or hypoplasia with a significantly higher frequency compared to the non-GDM group [15].

Several dedicated studies have been performed to understand the mechanisms behind the tooth development problem associated with DM. Abbassy et al. found that the mineral apposition rate in the incisors of type 1 diabetes (T1DM) rats is slower than in the control rats [9]. Gutowska et al. observed decreased fluorine concentrations in both teeth and serum, an increased calcium concentration in serum, and increased magnesium concentrations in both serum and teeth in T1DM rats, suggesting that abnormal mineral concentrations in the blood of DM animals affect the mineral composition of teeth, resulting in developmental defects of teeth [16]. Regarding the effect of GDM on fetal tooth development, Chen’s group utilized a GDM rat model and showed that the activation of TLR4/NF-ĸB signaling pathway in dental mesenchymal/epithelial cells and increased DNA methylation due to Apex1 downregulation in dental epithelial stem cells are involved in the defective tooth development in fetuses from GDM mothers [4,17,18].

The current study found that a murine model (*db/db* mouse) [19] of T2DM showed delayed enamel and dentin formation. The specific genes for pre-odontoblasts and pre-ameloblasts, as well as stem cell markers in the DPCs, were drastically decreased. In addition, cultured dental pulp cells derived from the T2DM mice showed decreased proliferation and differentiation compared with control dental pulp cells. Furthermore, the same cultured cells showed decreased stem/progenitor cell marker gene expression, as observed in vivo, suggesting that T2DM conditions impair the valuable characteristics of dental pulp cells in *db/db* mice.

## 2. Results

### 2.1. Delayed Development of Dentin and Enamel in T2DM Mouse Incisors

**Figure 1:**
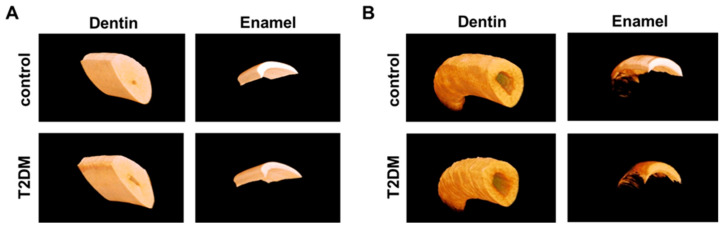
T2DM condition delays dentin and enamel formation in incisors. The 3D images produced with micro-CT data obtained from the mandibular incisors of control or T2DM mice. (**A**) The eruption site and (**B**) the apical site. The highly mineralized areas are displayed brightly.

To investigate the effect of the T2DM condition on tooth development, incisors and molars of control and T2DM mice (*Lepr^db^* mutant, *db/db*), which developed hyperglycemia after 4 weeks of age, were analyzed. Mandibular incisors from control and T2DM mice (10 weeks old) were analyzed using micro-CT (Figure 1). In the apical half of the incisors, enamel volume and density were significantly decreased by 55.7% and 20.3%, respectively, in T2DM mice compared to control mice, whereas the volume of dentin was reduced by 29.3% (Figure 1 and Table 1). In the coronal half, enamel density was decreased only by 6.5% in T2DM mice, and no change was observed in dentin (Figure 1 and Table 1). These results suggest that the T2DM condition causes delayed calcification and maturation in the dentin and enamel of the incisors, and the delay is almost recovered before eruption.

### 2.2. The Incisor Development Delay in db/db T2DM Mice Is Independent of the Mutation in Leptin Receptor

To eliminate the possibility that the delayed tooth development in *db/db* T2DM mice was caused by unexpected side effects associated with *db* mutation in the leptin receptor, the maxillary incisors from high-fat diet-induced obese (DIO) mice were micro-CT-scanned and compared to the controls. As expected, the incisors from DIO mice showed phenotypes remarkably analogous to those from T2DM mice (Table 2). In detail, the enamel volume and densities were decreased by 35.9% and 8.1%, respectively. Similarly, the dentin volume and densities were decreased by 27.6% and 7.6%, respectively. Notably, the densities of enamel and dentin were particularly decreased in the apical region (Table 2).

**Figure 2:**
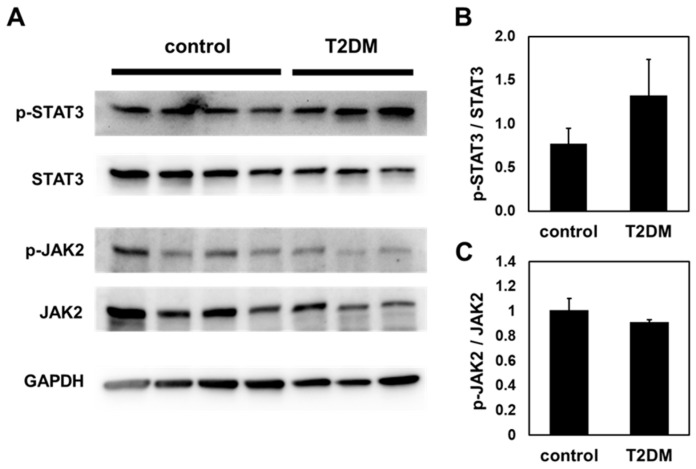
Quantitation of STAT3 and JAK2 phosphorylation in T2DM mouse incisors and the controls. (**A**) Images from the Western blots detecting phospho-STAT3, total STAT3, phospho-JAK2, total JAK2, and GAPDH in the protein samples from four control mouse incisors and three T2DM mouse incisors. (**B**) The relative ratio of phospho-STAT3 versus total STAT3 in the incisors from controls and T2DM mice. The graphed data were calculated from the averaged intensities of blot-(**A**). The *p*-value obtained with Student’s *t*-test comparing the two groups was 0.135. (**C**) The relative ratio of phospho-JAK2 versus total JAK2 in the incisors from controls and T2DM mice. The *p*-value obtained with Student’s *t*-test comparing the two groups was 0.132. The error bars indicate standard deviations.

Further, the activity of two major pathways driven by a leptin receptor (reviewed in [20,21,22]) was investigated. The phospho-STAT3 and the phospho-JAK2 amount relative to their total proteins from T2DM incisors and the controls were analyzed with a Western blot (Figure 2). Interestingly, the relative ratio of phospho-STAT3 and phospho-JAK2 was not statistically different between T2DM incisors and the controls (Figure 2B,C). These results suggest that the delay in the development of T2DM mouse incisors in the present study is independent of *db* mutation in the leptin receptor.

### 2.3. Transcriptomic Alteration in T2DM Mouse Incisors and the Alveoli

**Figure 3:**
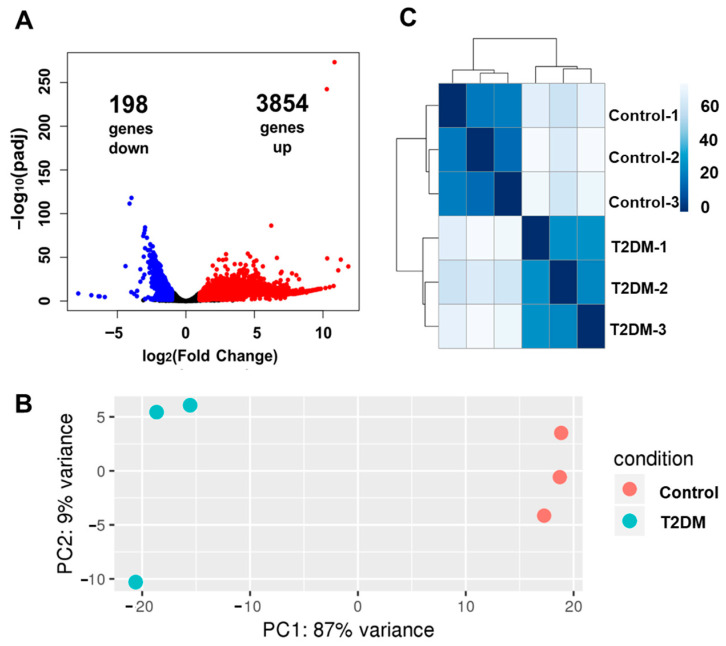
Visualized characteristics of the RNA-seq data from the incisors of control and T2DM mice. Total RNA(s) were isolated from mandibular incisors with the alveoli of three control and three T2DM mice at 10 weeks old. The Illumina HiSeq 2500 system was used to analyze transcript profiles. (**A**) Volcano Plot. The red and blue dots indicate genes showing more than 2-fold upregulation and downregulation, respectively. (**B**) Principal component (PC) analysis plot. (**C**) Heatmap of the sample-to-sample differences.

To investigate the molecular mechanisms of delayed dentin and enamel formation in T2DM incisors, a transcriptomic analysis of T2DM mouse incisors with their alveoli using RNA-seq was performed (Figure 3). A total of 4052 differentially expressed genes (DEGs) were observed in T2DM mice (*p* < 0.05; log Fold Change ± 2), including 3854 upregulated genes and 198 downregulated genes (Figure 3A). Principal component analysis (PCA) (Figure 3B) and the heatmap of the sample distance matrix (Figure 3C) revealed distinct variations between the expression profile of the control and T2DM samples; that is, the expected clustering of biological triplets was observed between control replications. Table 3 and Table 4 show the top 30 upregulated and downregulated genes, respectively. Interestingly, a group of homologous genes encoding protein with five transmembrane (5TM) domains showed the highest increase in the upregulated genes list (Table 3). These 5TM proteins are homologs of human PIRO (progranulin-induced receptor-like gene during osteoclastogenesis) protein [23], which was reported to have a relationship with osteoclast regulation, suggesting the possibility that T2DM condition affected osteoclast activities in alveoli. Meanwhile, the downregulated gene list showed a characteristic tendency with more than 10 muscle-related genes, such as *Mybpc2*, *Myh4*, and *Mylk2* (Table 4). These data could imply that muscle atrophy accompanied by T2DM (reviewed in [24,25]) was observed in the muscles adjunct to mandibular incisor alveoli, such as digastric, geniohyoideus, genioglossus, or a part of lateral masseter muscles. In addition, a gene ontology enrichment analysis was performed for the above differentially expressed genes; however, no gene group scored below 0.05 regarding the Benjamini–Hochberg false discovery rate (FDR). The lowest FDR was scored by GO:0006811 (ion transport genes) with FDR = 0.112.

### 2.4. Downregulated Odontoblast- and Ameloblast–Progenitor Specific Genes in T2DM Mouse Incisors

**Figure 4:**
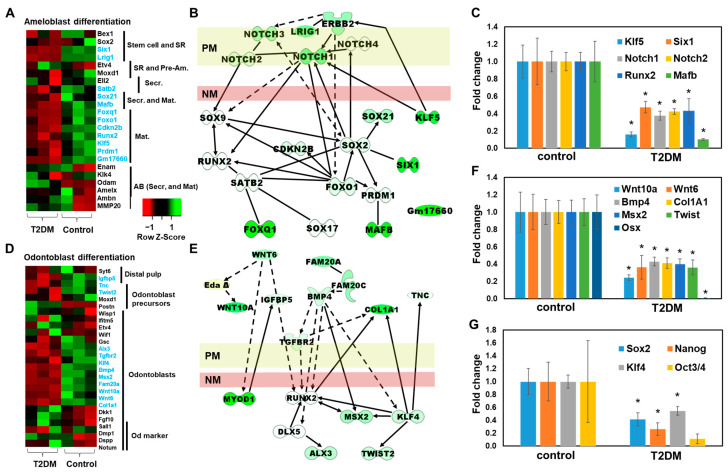
Decrease in progenitors and stem cell-specific gene expression in incisors of T2DM mice. Heatmaps of (**A**) ameloblast differentiation-related genes and (**D**) odontoblast differentiation-related genes, constructed from RNA-Seq data. Genes shown in blue are decreased in T2DM. Predicted pathways connecting (**B**) pre-ameloblast genes and (**E**) pre-odontoblast genes affected in T2DM condition. (**C**) Focused pre-ameloblast gene, (**F**) pre-odontoblast gene, and (**G**) stem cell gene expressions were quantified by qPCR. PM; plasma membrane. NM; nuclear membrane. * *p* < 0.05 with Student’s *t*-test.

To elucidate the delay of dentin/enamel development in T2DM mouse incisors, the RNA-seq data of ameloblast and odontoblast-specific genes [26,27] were assessed. Interestingly, secretion and mature ameloblast-specific genes (*Satb2*, *Sox21*, *Mafb*, *Foxq1*, *Foxo1*, *Cdkn2b*, *Runx2*, *Klf5*, *Prdm1*, Gm17660) and stem cell genes (*Six1*, *Lrig1*) were significantly downregulated in incisors of T2DM mice, whereas the major ameloblast specific genes such as *Enam*, *Klk4*, *Amelx*, *Ambn*, *Mmp20* were not changed (Figure 4A, Table 5 left). Using Ingenuity Pathway Analysis (IPA) (Qiagen), the Notch signaling pathway was predicted to correlate with the down-regulated genes (*Sox2*, *Sox21*, *Six1*, *Runx2*, *Foxq1*, *Foxo1*, *Mafb*) (Figure 4B). In the confirmation of these data by qPCR, the expressions of *Klf5*, *Six1*, *Notch2*, *Runx2*, and *Mafb* were significantly downregulated in incisors of T2DM mice by qPCR analysis (Figure 4C).

Likewise, odontoblast progenitor-specific genes (*Twist2*, *Igfbp5*, *Tnc*, *Gsc*, *Bmp4*, *Msx2*, *Klf4*, *Tgfbr2*, *Fam20a*) were also significantly downregulated in incisors of T2DM mice, while mature odontoblast markers did not decrease (Figure 4D, Table 5 right). From the IPA pathway analysis, the depression of Wnt and BMP signaling were inferred to correlate with attenuation of odontoblast proliferation and differentiation (Figure 4E). From qPCR analysis, the gene expressions of *Wnt10a*, *Wnt6*, *Bmp4*, *Col1A1*, *Msx2*, *Twist1*, and *Osx* (*Sp7*) were significantly decreased in incisors of T2DM mice (Figure 4F), while major odontoblast specific genes (*Dmp1*, *Dspp*, *Nortum*) were not significantly changed, which was a similar phenomenon to ameloblast specific genes described above. In addition, general stem cell markers such as *Sox2*, *Nanog*, *Klf4*, and *Oct3/4* were reduced in incisors of T2DM mice compared to control mice (Figure 4G), suggesting that T2DM condition impaired the activity of progenitors and stem cells in incisors.

### 2.5. Cultured DPCs from T2DM Mice Inherit Impaired Characteristics Observed In Vivo

**Figure 5:**
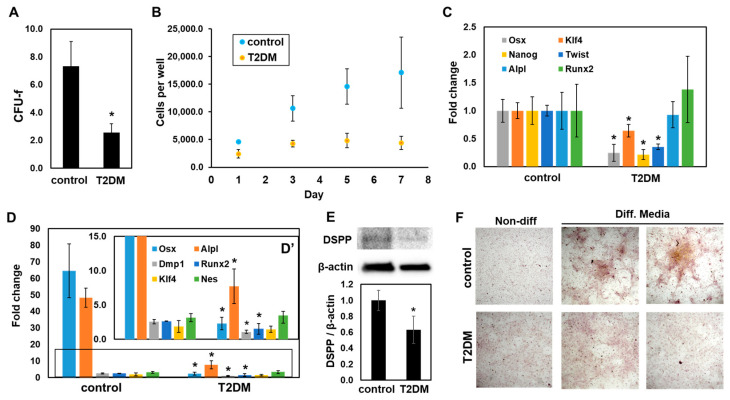
Diminished cell proliferation and differentiation in T2DM DPC. (**A**) Fibroblastic colony-forming units (CFU-F) of dental pulp cells (DPCs) from T2DM and the control mice. The 2 × 10^3^ DPCs were cultured for 14 days and then fixed with 4% paraformaldehyde. Following the staining with 0.5% crystal violet, the colony numbers were counted and used for CFU-F calculation. All data here are averaged from triplicate cultures, and the error bars and asterisks indicate standard deviation and *p* < 0.05 data in Student’s *t*-test, respectively, unless noted. (**B**) Growth curve of DPCs from T2DM and control mice. The 2 × 10^3^ DPCs were seeded in 96-well plate and cultured for up to 7 days. The cells isolated with trypsinization were counted every other day. (**C**,**D**) qPCR analysis of (**C**) stem cell-related genes and (**D**) odontogenic differentiation-related genes in cultured DPCs. (**D**) DPCs were cultured in differentiation condition media for up to 21 days, and the odontoblast-specific gene expressions were analyzed with qPCR. Boxed area is magnified in (**D′**). (**E**) The DSP amount of the differentiated DPCs used in (**D**) was analyzed with Western blot. The DSP signal strength normalized by β-actin signal is shown as a graph at the bottom. (**F**) Mineralization of the differentiated DPC. DPCs were stained with Alizarin red, following the same treatment as in (**C**). The images were taken by a phase-contrast inverted light microscope (Nikon) with 100× magnification.

The characteristics of dental pulp cells isolated from control and T2DM mice were investigated. The numbers of colony-forming unit–fibroblast (CFU-f) were reduced in dental pulp cells (DPC) isolated from T2DM incisors compared to the control incisors (Figure 5A). In addition, the growth curve of dental pulp cells in T2DM mice was significantly decreased, whereas the doubling time of T2DM DPC (2.46 days) was much higher than control DPC (1.656 days) (Figure 5B). Interestingly, gene expressions of pre-odontoblast and the progenitor-specific genes, such as *Osx*, *Klf4*, *Nanog*, and *Twis*t1, were significantly reduced in cultured T2DM DPC, whereas *Alpl* and *Runx2* were not changed (Figure 5C). These data suggest that the stemness of DPCs from T2DM mice is irreversibly attenuated.

In osteo/odontogenic differentiation assay using the cultured DPCs from T2DM and the controls, odontoblast-specific genes, *Alpl*, *Dmp1*, *Osx*, and *Runx2* were significantly decreased in T2DM DPC compared to control DPC, whereas gene expression of *Klf4* and *Nes* was not altered (Figure 5D,D’). Additionally, dentin sialo protein (DSPP) in differentiated DPC from T2DM decreased by about 40% (Figure 5E). T2DM DPCs showed less mineral deposition compared to control DPCs (Figure 5F). These results indicate that T2DM DPCs have impaired odontogenic differentiation potential compared to WT DPCs.

Taken together, the above data suggest that the tooth development delay in T2DM mice was correlated with the attenuated characteristics of stem/progenitor cells.

## 3. Discussion

### 3.1. Delay of Incisor Development

**Figure A1:**
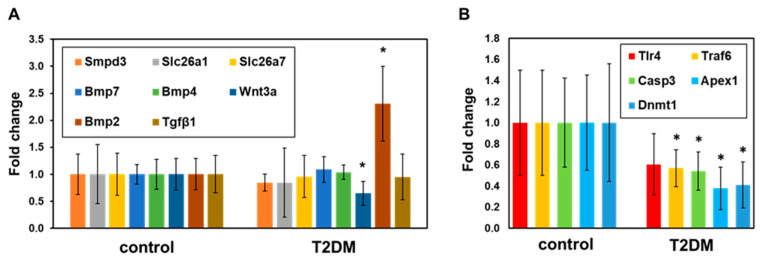
qPCR analysis using RNAs from incisors of T2DM mice and the controls, comparing the expression of the genes (**A**) previously known to be related to delayed tooth/dentin development [28,29,30,31,32] and the genes (**B**) found to affect the tooth development of the children from GDM mothers [4,17,18]. Values are averaged from seven control and eight T2DM samples, respectively. * *p* < 0.05 with Student’s *t*-test.

In the present study, delayed development of dentin and enamel in T2DM mouse incisors was observed. Similar delays of incisor development have been observed in mice with some genetic modification (summarized in Table 6), such as *Slc26a1* and *Slc26a7* deletion [28], *Smpd3* knockout [29], *Bmp7* knockout in neural crest [30], NOTCH1/2 signaling inhibition [31], and *Stat3* knockout [32]. Among those genes, the expressions of *Bmp7*, *Smpd3*, *Slc26a1*, and *Slc26a7* did not change in our *db/db* mouse incisor in RT-qPCR assay (Figure A1A), indicating that the delay of incisor development in the present study should be independent of transcriptional change in these genes. Also, *Stat3* has already been excluded from the causal factor of the delay in Section 2.2 above.

On the other hand, the expression of *Notch1/2* and Wnt genes showed various alterations in T2DM mouse incisors (Figure 4C,F, Figure A1A). As NOTCH1/2 inhibition in mouse incisor is reported to attenuate the desmosome structure in ameloblasts, resulting in poor enamel formation [31], investigation of desmosome structure in T2DM mice will be helpful to verify if the same machinery is delaying the enamel formation. Further, analyzing every Wnt pathway will be of significant importance in future studies.

Delayed incisor development has also been reported in research using mice with hypophosphatasia (HPP) [33,34,35,36], dyslipidemia caused by LDL receptor deficiency [37], and hyperlipidemia [38]. However, in these reports, the enamel development delays observed in the current study were not found. Further, dyslipidemia and hyperlipidemia mouse incisors show significantly thicker dentin with narrowed pulp space compared to the wild type, which was not observed in the current study. Thus, those studies reporting delayed incisor development have characteristics different from our present report, suggesting that our finding contains some machinery independent from those reports.

**Table 6:** Dental-defect rodent models discussed in this study.

The Dental Defects Cited in This Study	Authors	Causal Factors
Delayed development	Millan et al., 2008 [33]	Hypophosphatasia
	Foster et al., 2013 [35]	Hypophosphatasia
	Khavandgar et al., 2013 [29]	*Smpd3* knockout
	Jheon et al., 2016 [31]	NOTCH1/2 signaling inhibition
	Ye et al., 2016 [38]	Hyperlipidemia
	Yin et al., 2017 [28]	*Slc26a1* and *Slc26a7* deletion
	Zhang et al., 2018 [32]	*Stat3* knockout
	Kurotaki et al., 2020 [37]	Dyslipidemia caused by LDL receptor deficiency
	Malik et al., 2020 [30]	*Bmp7* knockout in neural crest
More wear	Atar et al., 2007 [39]	T2DM
Dentin/enamel hypoplasia	Abbassy et al., 2015 [9]	T1DM
Reduced dentin/enamel microhardness	Saghiri et al., 2022 [40]	T1DM
Suppressed enamel formation	Chen et al., 2017 [4]	T1DM GDM
Suppressed dentin formation	Lyu et al., 2020 [18]	T1DM GDM

### 3.2. Differentially Expressed Genes (DEGs) in T2DM Incisor

In the top 30 upregulated genes in T2DM incisors, 5TM genes homologous to human PIRO genes showed the greatest increase (Table 3). Some genes in this group, such as Gm10715, appear in the DEG list in a variety of the literature, which describes the whole-body reaction during influenza infection [41], the high-fat diet [42], or the developing intestinal epithelium under antibiotics [43]. Importantly, the expression of Gm10800, one of such a gene on the list, is reported to be essential for multinucleated osteoclast formation and is strongly induced by progranulin and RANKL in macrophage [23], implying that the other PIRO homologs on the list may have a similar function. This suggests that osteoclast activity could be augmented in T2DM mouse incisor alveolus, which matches the well-reported fact that diabetes complicates periodontitis through osteoclast activity (reviewed in [44]). Likewise, the downregulation of skeletal muscle genes in our T2DM mice (Table 4) mirrors the phenomena of muscle atrophy observed in diabetes patients (reviewed in [24,25]). Taken together, the largest group of the above DEGs agreed well with known diabetic complications; however, those genes may not explain the delayed incisor development in T2DM mice because those genes are thought to be expressed outside of the incisors.

### 3.3. Stem Cell Abnormality and Differentiation Defect of Progenitor Cells in T2DM

In the present study, stem cell marker gene expressions were attenuated in dental pulp from T2DM mice. Stem cell abnormalities caused by T2DM have been reported across a variety of sources (reviewed in [45]). A report about the depression of osteoblastic potential of mesenchymal stem cells [46] would be especially relevant to the current study, as osteoblasts are thought to be analogs of odontoblasts regarding their osteo/odontogenic differentiation. According to the report, sera from T2DM patients were applied to mesenchymal stem cell (MSC) culture, and it was found that osteogenic genes, *Runx2*, OPN (*Spp1*), and OCN (*Bglap*) gene expressions in the MSCs were depressed with the increasing level of glycosylated hemoglobin A1c in the sera [46]. Recently, an Indian group found that osteogenic and chondrogenic differentiation abilities were attenuated in human DPSCs from diabetic donors, while adipogenic differentiation of those cells was significantly higher than DPSCs from healthy donors [47]. These reports regarding the decreased osteogenic differentiation ability of stem cells from diabetic patients parallel and support the current findings in T2DM mice. Notably, in the current experiments, abnormalities of stem cells in T2DM mice were inherited in the cultured DPCs (Section 2.5). This suggests that the abnormalities of stem cells could be caused by epigenetic changes in the genome; however, the type of causative epigenetic changes has not been identified (see Section 3.4).

### 3.4. Tooth Development in Diabetic Patients

The effects of diabetic conditions on tooth development have been extensively studied in rodent teeth as a model of human diabetic patients (summarized in Table 6). A 3-D analysis of T2DM mice using X-ray microtomography (XMT) showed more wear in their teeth [39]. The micro-CT analysis of T1DM mice found dentin/enamel hypoplasia and a significant reduction in the dentin mineral apposition rate in their incisors [9]. T1DM condition also negatively affects the microhardness of dentin and, especially, the enamel of their incisors [40]. Mechanistic explanations for these observations are strongly awaited to elucidate dental problems in diabetic patients.

In humans, the most evident influence of T2DM on developing teeth occurs in the fetuses of pregnant women with GDM or PDM. Recently, a series of rat studies to analyze tooth development by simulating human GDM conditions were performed by Chen’s group [4,17,18]. These studies showed that the T1DM condition of pregnant mother rats affects several pathways in the offspring’s tooth germs. (1) TLR4/NF-κB signaling pathways in dental mesenchymal/epithelial cells in T1DM rats are activated, which results in the aggravation of proliferation and the apoptosis of those cells, as well as the inhibition of SMAD1/5/8 phosphorylation leading to decreased odontoblasts differentiation and dentin formation. (2) The DNA methylation in the dental epithelial stem cell increases due to the Dnmt1 upregulation caused by the downregulated Apex1 expression, resulting in the suppression of their proliferation and impaired incisor enamel formation.

In comparison to the above studies from Chen’s group, the current study observed different behaviors of the TLR4–apoptosis pathway or DNA methylation in adult T2DM mouse incisors (Figure A1B). In detail, a significantly increased expression of *Tlr4* or *Traf6* genes reported to be upregulated in the papers from Chen’s group was not observed, but a decreased *Casp3*, a major apoptosis marker, was observed with qPCR and RNA-seq (Figure A1B). Regarding DNA methylation, although a decreased *Apex1* expression was observed, *Dnmt1* expression did not decrease in T2DM mice (Figure A1B). These differences could be due to the diverse characteristics between developing teeth in fetuses and growing incisors in adult animals. Also, Chen’s GDM model is T1DM produced by injecting a high dose of streptozotocin into pregnant rats [17], while the mouse model used in the current study is *db/db* T2DM [19]. To overcome differences in these conditions, the most straightforward solution will be the utilization of the T2DM GDM mouse model.

## 4. Materials and Methods

### 4.1. Animals

All mice were housed by 2~5 animals per cage with free access to food and water in a facility with 12-hour light–dark cycles. The control (Dock7^m^+/Dock7^m^+), T2DM (*db/db*, BKS.Cg-Dock7^m^+/+, *Lepr^db^*/J) C57BL/6J mice (#000697), and diet-induced obese (DIO) C57BL/6J mice (#380050) were purchased from Jackson laboratory. These animals were housed following the guidelines provided by the Rutgers University IACUC. Animals were weighed periodically and monitored for blood glucose levels (non-fasting). DIO mice were fed a high-fat diet (35 g%, Research Diet Inc., New Brunswick, NJ, USA) for eight weeks. Ten-week-old T2DM (*db/db*), control, and DIO mice were euthanized with carbon dioxide, followed by a necropsy to collect maxilla and mandibular.

### 4.2. Micro-CT Analysis

Mandibles from five control and five T2DM mice and maxillae from five control and five DIO mice were isolated and stored in 70% ethanol at 4 °C until processing. They were scanned by the micro-CT instrument, Skyscan 1172, at 70 kV, 7.45 mm per pixel, at 0.3-degree rotation. The machine was calibrated using phantoms with CaHA concentrations of 0.25 and 0.75 g/cm^3^. Reconstruction of raw images was performed using NRecon V1.4.0 software (Skyscan, Bruker, Billerica, MA, USA). Obtained images were analyzed using CTan Version 1.18.4.0+ (CT-vol software, Bruker, Billerica, MA, USA). The 3D images were generated by CTvox (Skyscan, Bruker, Billerica, MA, USA). For the mandibular incisors, the transverse slides from the apical end to the mesial side of the first molar and from the mesial side of the first molar to the erupted site of the alveolar bone were used for analysis [48]. For the maxillary incisors, the transverse slides from the apical end to the posterior edge of the eruption site and those apical halves were used for analysis.

### 4.3. RNA Isolation and qPCR

Total RNA was isolated from mandibular incisors with their alveoli from four to five mice of each group (control and T2DM) using Direct-zol RNA Miniprep Plus (Zymo Research, Irvine, CA, USA). The cDNA was synthesized from at least 500 ng of isolated RNA using a High-Capacity cDNA Reverse Transcription Kit (Thermo Fisher Scientific, Waltham, MA, USA), following the manufacturer’s instructions. qPCR was performed using PowerUp™ SYBR^®^ Green Master Mix (Thermo Fisher Scientific) and 200 pM gene-specific primers (Table A1). The thermal cycler condition was 40 cycles (95 °C for 15″, 60 °C for 30″ and 72 °C for 30″). All qPCR data shown in this study were obtained from the average of at least three independent samples. The error bars indicate the standard deviation. All statistical analyses in this study were performed using the Student’s *t*-test, assuming unpaired two-tailed data distribution.

### 4.4. Cells and Culture Conditions

Dental pulp cells (DPCs) were isolated from the incisors or molars of three mice from each group (control and T2DM). DPCs were minced in phosphate-buffered saline (PBS) and incubated in HBSS containing 3U collagenase P (Sigma-Aldrich, St. Louis, MO, USA) for 15 min at 37 °C, followed by culturing in alpha-MEM with 20% fetal bovine serum, 2 mM L-glutamine, and 1× Antibiotic–Antimycotic (Thermo Fisher Scientific) at 37 °C in 5% CO_2_.

For DPC differentiation, DPCs were cultured with differentiation media containing alpha-MEM (as above) plus 10 ng/mL transforming growth factor beta-1 (TGFβ-1), 10 nM dexamethasone, 50 μg/mL L-ascorbic acid, and 10 mM beta-glycerophosphate (Sigma-Aldrich) for up to 21 days. After differentiation, cells were fixed with 4% paraformaldehyde, stained with Alizarin red solution, washed three times with distilled water, and microscopically analyzed.

### 4.5. Western Blot Analysis

Analysis was conducted on cell lysates from cultured DPC isolated from incisors or minced incisors of three to four mice from each group (control and T2DM). Protein lysates were prepared by incubating the cells for 15 min at 4 °C in RIPA buffer, Halt™ Protease, and Phosphatase Inhibitor Cocktail (Thermo Fisher Scientific) prior to centrifugation at 13,000 rpm for 10 min at 4 °C. Total protein was quantified by a Bradford assay (Bio-Rad Laboratories, Hercules, CA, USA). Proteins were used for sodium dodecyl sulfate–polyacrylamide gel electrophoresis (SDS-PAGE) on a 4–15% gradient polyacrylamide gel (Mini-PROTEAN, Bio-Rad, Hercules, CA, USA), and the migrated proteins were transferred to polyvinylidene difluoride (PVDF) membrane. To block non-specific protein binding, the membrane was incubated in 5% non-fat dry milk at room temperature, prior to overnight incubation at 4 °C with primary antibodies raised against DSPP (sc-73632, Santa Cruz Biotechnology, Dallas, TX, USA), β-actin (#4967, Cell Signaling Technology, Danvers, MA, USA), phospho-STAT3 (#9145, Cell Signaling Technology), total STAT3 (#9139, Cell Signaling Technology), phospho-JAK2 (#3776, Cell Signaling Technology), total JAK2 (#3230, Cell Signaling Technology), or GAPDH (#5174, Cell Signaling Technology).

### 4.6. RNA-Sequencing (RNA-Seq) and Data Analysis

Three incisors from each mouse group (control and T2DM) were homogenized with BeadBug™6 and total RNA using Direct-zol RNA Miniprep Plus (Zymo Research, Irvine, CA, USA) and quantified spectrophotometrically (Nanodrop 2000, Thermo Fisher Scientific). RNA-seq was performed in Azenta Life Sciences, and the obtained raw data were exported to Strand-NGS ver4.0 (Strand Life Sciences, Bangalore, India) for subsequent analysis. The Illumina HiSeq 2500 system (Illumina, Inc., San Diego, CA, USA) was used to analyze the transcript profiles of the incisors of control and T2DM mice at 10 weeks old. The RNA-seq analysis was performed on three independent biological samples for each genotype (*n* = 3) with a 2 × 150 bp configuration and single index per lane in a paired-end experimental design. Genes that were differentially expressed (>2.0 fold) in the T2DM group relative to control were identified after passing a *t*-test (*p* < 0.05) and post-hoc test (Storey with Bootstrapping) with a corrected q-value of 0.05. Genes in the expression data sets were first “ranked” based on Log2 values from highest to lowest for both groups at both time points prior to hierarchical clustering being used to group gene expression in each condition using the default settings in Strand-NGS.

## 5. Conclusions

Diabetes mellitus is a systemic disease on the rise worldwide, which adversely affects various aspects of oral health. The current study started with the hypothesis that the mechanism of adverse effects of T2DM on tooth development can be elucidated through transcriptomic approaches. As a result, delayed tooth development and attenuated characteristics of stem/progenitor cells in T2DM mouse incisors were observed. These observations may contribute to the understanding of the DM’s effects on tooth development. However, utilizing incisors from genetically modified rodents and the lack of in vivo demonstration experiments using some reagent to modulate stem cell function can be pointed out as limitations of the current study. These limitations could be overcome by exploiting a fat-diet-induced GDM mouse model and local injection of reagents, e.g., Fisetin [49], to delay the differentiation of dental stem cells. The implementation of these plans, along with the development of this area of research, will foster a more realistic understanding of the effects of DM on tooth development in humans, which will significantly contribute to the oral health of DM patients worldwide.

## Figures and Tables

**Table 1:** The micro-CT data of mandibular incisors from control and T2DM mice.

	Control ^a^	T2DM ^a^	*p*-Value ^b^	% Change
** Apical side**				
Dentin volume	0.8285 (0.0694)	0.5858 (0.0146)	0.0131	−29.3
Dentin density	1.4582 (0.0267)	1.4536 (0.0342)	0.9172	−0.3
Enamel volume	0.2729 (0.0119)	0.1208 (0.0207)	<0.0001	−55.7
Enamel density	2.0432 (0.1115)	1.6294 (0.0979)	0.0138	−20.3
Pulp volume	0.5517 (0.0331)	0.5523 (0.0437)	0.7311	0.1
** Tip side**				
Dentin volume	1.4266 (0.2062)	1.4964 (0.1351)	0.3920	4.8
Dentin density	1.4544 (0.0554)	1.4064 (0.0907)	0.1797	−3.3
Enamel volume	0.2929 (0.0364)	0.2826 (0.0212)	0.4501	−3.5
Enamel density	2.7739 (0.1144)	2.5938 (0.1215)	0.0003	−6.5
Pulp volume	0.0943 (0.0567)	0.1041 (0.0614)	0.7301	10.3

^a^ Averaged volume (mm^3^) and mineral density (g/cm^3^) from five samples. Values in parentheses are standard deviations. ^b^ The *p*-values are calculated using two-tailed Student’s *t*-test. *p*-values < 0.05 and the %changes are indicated in red.

**Table 2:** The micro-CT data of maxillary incisors from control and DIO mice.

	Control ^a^	DIO ^a^	*p*-Value ^b^	% Change
** Total ^c^**				
Dentin volume	1.2434 (0.1745)	0.9024 (0.1098)	0.0082	−27.6
Dentin density	1.4771 (0.0445)	1.3644 (0.0253)	0.0023	−7.6
Enamel volume	0.2460 (0.0462)	0.1577 (0.0201)	0.0094	−35.9
Enamel density	2.0830 (0.0485)	1.9131 (0.0361)	0.0003	−8.1
** Apical region ^d^**				
Dentin density	1.3264 (0.0706)	1.1555 (0.0347)	0.0031	−12.9
Enamel density	1.8814 (0.1039)	1.5038 (0.1055)	0.0005	−20.1

^a^ Averaged volume (mm^3^) and mineral density (g/cm^3^) from five samples. Values in parentheses are standard deviations. ^b^ The *p*-values are calculated using two-tailed Student’s *t*-test. *p*-values < 0.05 and the %changes are indicated in red. ^c^ These values were collected from the area of maxillary incisor between the apical end and the posterior edge of eruption site. ^d^ The region here corresponds to the apical half of maxillary incisor between the apical end and the posterior edge of eruption site.

**Table 3:** The top 30 upregulated genes in incisors and the alveolus of T2DM mice compared to the control mice.

Gene ID	Base Mean	Log2 FoldChange	Adjusted *p*-Value	Description
Gm10715	1260.3	11.8	2.72 × 10^−^^40^	predicted to encode protein with 5 TM domains
Gm10720	1146.9	11.3	3.69 × 10^−^^48^	predicted to encode protein with 5 TM domains
Gm17535	756.6	11.1	8.11 × 10^−^^36^	predicted to encode protein with 5 TM domains
Gm11168	8573.9	10.8	<1.00 × 10^−^^100^	predicted to encode protein with 5 TM domains
Gm37985	215.0	10.8	6.22 × 10^−^^18^	lncRNA predicted to target Hif3a, a protein reguling adaptive responses to hypoxia
Gm10800	1,357,771.3	10.7	<1.00 × 10^−^^100^	predicted to encode protein with 5 TM domains
Gm21738	153,254.1	10.6	<1.00 × 10^−^^100^	predicted to encode protein with 5 TM domains
Gm26870	223,896.6	10.6	<1.00 × 10^−^^100^	lncRNA related to osteoclast function
Gm10801	149,821.0	10.6	<1.00 × 10^−^^100^	predicted to encode protein with 5 TM domains
Gm10717	16,702.3	10.6	<1.00 × 10^−^^100^	predicted to encode protein with 5 TM domains
AW822073	90.7	10.5	1.23 × 10^−^^16^	predicted DNA-binding transcription factor
Gm10722	27,085.4	10.5	<1.00 × 10^−^^100^	predicted to encode protein with 5 TM domains
Gm10718	15,183.5	10.4	<1.00 × 10^−^^100^	predicted to encode protein with 5 TM domains
Gm10721	721.8	10.3	1.61 × 10^−^^49^	predicted to encode protein with 5 TM domains
Gm10719	5665.1	10.3	<1.00 × 10^−^^100^	predicted to encode protein with 5 TM domains
H2-Ea-ps	72.4	10.2	1.46 × 10^−^^15^	pseudogene of an MHC class II antigen
Apob	58.3	9.8	9.74 × 10^−^^15^	apolipoprotein B
Scn10a	55.3	9.8	1.22 × 10^−^^14^	voltage-gated sodium channel subunit (NaV1.8)
Slc6a5	52.8	9.7	2.22 × 10^−^^14^	sodium- and chloride-dependent glycine transporter
Scn9a	52.5	9.7	2.19 × 10^−^^14^	voltage-gated sodium channel subunit (NaV1.7)
Duxf3	99.3	9.7	1.83 × 10^−^^14^	double homeobox family member 3
Muc5ac	92.7	9.6	9.41 × 10^−^^14^	mucin 5AC
BC048546	47.4	9.6	1.02 × 10^−^^13^	ovostatin homolog
Gm29154	47.1	9.5	1.12 × 10^−^^13^	ncRNA
Abca17	45.2	9.5	1.22 × 10^−^^13^	ATP-binding cassette, subfamily A (ABC1), member 17
Mroh2b	45.2	9.5	1.48 × 10^−^^13^	maestro heat-like repeat-containing protein, may relate to sperm capacitation
Spag17	44.1	9.5	1.14 × 10^−^^13^	sperm associated antigen 17
Gm4981	42.6	9.4	1.79 × 10^−^^13^	predicted DNA-binding transcription factor
Dcdc2a	42.1	9.4	1.99 × 10^−^^13^	doublecortin domain-containing protein involved in neuronal migration
Slc7a14	39.7	9.3	5.23 × 10^−^^13^	solute carrier family involved in amino acid transport

**Table 4:** The top 30 downregulated genes in incisors and alveoli of T2DM mice compared to the control mice. Muscle/myocyte-related genes are indicated with asterisks.

Gene ID	Base Mean	Log2 FoldChange	Adjusted *p*-Value	Description
Nlrp1c-ps	30.57	−7.85	2.26 × 10^−^^9^	pseudogene related to the nod-like receptor (NLR) family
mt-Tg	15.74	−6.90	2.3 × 10^−^^7^	mitochondrial tRNA for glycine
6820402A03Rik	10.93	−6.37	3.81 × 10^−^^6^	RIKEN cDNA
RP24-286J14.3	10.35	−6.29	4.27 × 10^−^^6^	predicted gene
RP24-286J14.2	7.97	−5.91	2.61 × 10^−^^5^	predicted gene
AI506816	177.06	−4.40	1.30 × 10^−^^40^	lncRNA related to wounding. Also expressed in embryo
Mybpc2	9455.72	−4.11	<1.00 × 10^−^^100^	myosin Binding Protein C2 *
9930111J21Rik2	35.86	−3.98	6.37 × 10^−^^11^	Interferon-gamma-inducible GTPase
Actn3	5074.53	−3.97	<1.00 × 10^−^^100^	alpha-actinin-3 *
Myh4	39,961.13	−3.77	6.64 × 10^−^^9^	myosin Heavy Chain 4 *
RP23-33N12.2	18.04	−3.57	2.83 × 10^−^^6^	predicted gene
Gm22513	42.81	−3.55	2.23 × 10^−^^12^	snRNA
Nat8l	950.59	−3.32	4.38 × 10^−^^37^	N-acetyltransferases, involved in cell adhesion
5830417I10Rik	95.85	−3.32	7.80 × 10^−^^23^	gon-4-like pseudogene, predicted to be a transcription factor
Dupd1	184.54	−3.12	4.34 × 10^−^^27^	phosphatase involved in protein dephosphorylation and MAPK inhibition
Scn1b	1723.75	−3.11	4.27 × 10^−^^75^	voltage-gated sodium channel *
Ankrd23	1137.36	−3.07	1.79 × 10^−^^51^	transcriptional regulator *
Hfe2	1437.16	−3.05	2.85 × 10^−^^78^	hemojuvelin, involved in iron metabolism and regulation of hepcidin expression
Mir6236	387.09	−3.02	6.56 × 10^−^^31^	microRNA, potentiates adipocyte insulin signaling
Jph2	1343.26	−3.00	8.79 × 10^−^^82^	junctophilin-2, a membrane protein essential for cardiac myocytes *
Tmem267	8.61	−2.98	3.17 × 10^−^^3^	possible oncogene encoding a transmembrane protein
Smtnl2	1171.51	−2.98	3.58 × 10^−^^61^	smoothelin-like 2, a protein involved in actin cytoskeleton organization
Mylk2	1573.04	−2.98	6.04 × 10^−^^85^	myosin Light Chain Kinase 2 *
Mir1247	8.56	−2.96	5.12 × 10^−^^3^	microRNA involved in post-transcriptional regulation of SOX93 in cartilage
Pygm	9862.36	−2.84	4.20 × 10^−^^73^	myophosphorylase, an enzyme that breaks down glycogen *
Aldoa	26,984.31	−2.76	5.82 × 10^−^^59^	aldolase A, a glycolytic enzyme that convert F1,6BP to G3P and DHAP
Kcna7	345.70	−2.75	2.90 × 10^−^^45^	voltage-gated potassium channel subunit *
Gm12953	17.46	−2.75	5.15 × 10^−^^5^	predicted gene
Jsrp1	519.53	−2.72	2.09 × 10^−^^45^	Junctional Sarcoplasmic Reticulum Protein 1 *
Ky	861.07	−2.68	2.24 × 10^−^^45^	kyphoscoliosis peptidase *

**Table 5:** RNA-seq data of ameloblast-related genes (left) and odontoblast-related genes (right) used for IPA. Significant changes (*p* < 0.05) or reductions of more than 2-fold are shown in yellow.

Gene ID	Base Mean	Log2 Fold Change	Adjusted *p*-Value	Gene ID	Base Mean	Log2 Fold Change	Adjusted *p*-Value
Bex1	24.7	−0.5	2.77 × 10^−1^	Syt6	140.0	−0.2	4.91 × 10^−1^
Sox2	39.7	−0.8	8.90 × 10^−2^	Igfbp5	8221.2	−0.8	1.14 × 10^−6^
Six1	466.7	−1.7	5.17 × 10^−24^	Tnc	6862.4	−0.7	3.72 × 10^−5^
Lrig1	476.3	−1.3	3.60 × 10^−16^	Postn	15,375.8	2.2	3.35 × 10^−39^
Etv4	55.2	0.4	2.60 × 10^−1^	Twist2	48.4	−1.0	4.86 × 10^−3^
Moxd1	189.7	−0.3	1.53 × 10^−1^	Moxd1	189.7	−0.3	1.53 × 10^−1^
Ell2	320.1	−0.2	3.18 × 10^−1^	Wisp1	1364.8	0.3	9.93 × 10^−2^
Satb2	1471.5	−0.6	1.22 × 10^−3^	Ifitm5	46.3	0.3	4.70 × 10^−1^
Sox21	120.9	−1.1	6.37 × 10^−4^	Etv4	55.2	0.4	2.60 × 10^−1^
Mafb	1045.6	−1.8	3.08 × 10^−13^	Wif1	1095.4	0.3	6.31 × 10^−2^
Foxq1	259.8	−2.5	1.48 × 10^−23^	Gsc	23.4	−0.6	1.77 × 10^−1^
Foxo1	562.4	−0.8	5.59 × 10^−5^	Alx3	194.0	−1.1	3.40 × 10^−6^
Cdkn2b	134.4	−0.9	8.14 × 10^−5^	Tgfbr2	1386.9	−0.9	8.39 × 10^−9^
Runx2	1355.1	−0.6	5.75 × 10^−4^	Klf4	449.3	−1.0	3.35 × 10^−5^
Klf5	268.6	−1.7	1.94 × 10^−3^	Bmp4	227.1	−1.0	2.63 × 10^−7^
Prdm1	201.7	−0.6	5.28 × 10^−3^	Msx2	269.8	−1.1	2.02 × 10^−8^
Gm17660	345.4	−1.3	5.28 × 10^−11^	Fam20a	549.4	−1.3	3.60 × 10^−12^
Enam	2062.8	0.8	4.22 × 10^−1^	Wnt10a	272.4	−1.5	8.96 × 10^−14^
Klk4	194.0	0.4	7.64 × 10^−1^	Dkk1	71.2	0.9	4.41 × 10^−3^
Odam	4713.5	0.8	5.76 × 10^−7^	Fgf10	27.7	1.6	6.79 × 10^−4^
Amelx	6204.2	0.1	7.56 × 10^−1^	Wnt6	146.8	−1.1	1.40 × 10^−5^
Ambn	13,259.4	0.1	5.33 × 10^−1^	Col1a1	256,336.4	−1.6	3.94 × 10^−21^
Mmp20	595.9	0.8	2.50 × 10^−1^	Sall1	58.6	0.7	2.58 × 10^−2^
				Dmp1	1683.6	−0.1	4.74 × 10^−1^
				Dspp	12,986.9	0.3	9.59 × 10^−2^
				Notum	46.8	0.6	1.74 × 10^−1^

## Data Availability

The original RNA-seq data presented in this study are openly available in the next NCBI URL upon acceptance of the current manuscript: https://dataview.ncbi.nlm.nih.gov/object/PRJNA1191555?reviewer=gkm01nuhb5sm2go8t0li5q70ec (accessed on 29 November 2024). Accession number: PRJNA1191555 (Release date 31 January 2025). All other data presented in this study are included in the article. Further inquiries can be directed to the corresponding author.

## Appendix A

**Table A1:** Primers used for RT-qPCR. All sequences are aligned from the 5′ to 3′ position.

Gene/Primers	Sequences	Gene/Primers	Sequences
mALPL-F	CCAGAAAGACACCTTGACTGTGG	mOsx-F	TTCTGCGGCAAGAGGTTCACTC
mALPL-R	TCTTGTCCGTGTCGCTCACCAT [50]	mOsx-R	GTGTTTGCTCAGGTGGTCGCTT [51]
mBmp2-F	AACACCGTGCGCAGCTTCCATC	mRunx2-F	CCTGAACTCTGCACCAAGTCCT
mBmp2-R	CGGAAGATCTGGAGTTCTGCAG [52]	mRunx2-R	TCATCTGGCTCAGATAGGAGGG [53]
mBmp4-F	GCCGAGCCAACACTGTGAGGA	mSix1-F	AGGTCAGCAACTGGTTTAAGAACC
mBmp4-R	GATGCTGCTGAGGTTGAAGAGG [54]	mSix1-R	GAGTTGATTCTGCTTGTTGGAGG [55]
mBmp7-F	GGAGCGATTTGACAACGAGACC	mSlc26a1-F	GATCATTGGGCTACAGGACTGC
mBmp7-R	AGTGGTTGCTGGTGGCTGTGAT [54]	mSlc26a1-R	GAGAGATAGGTAGACACGAAGCC [56]
mCol1a1-F	CCTCAGGGTATTGCTGGACAAC	mSlc26a7-F	CTCTCGCACAAGGATCTGCCAA
mCol1a1-R	CAGAAGGACCTTGTTTGCCAGG [50]	mSlc26a7-R	ACAAACCAGCCGTCCTTCCCAT [56]
mDmp1-F	AGTGAGTCATCAGAAGAAAGTCAAGC	mSmpd3-F	TCAACAGCGGTCTCTTCTTCGC
mDmp1-R	CTATACTGGCCTCTGTCGTAGCC [57]	mSmpd3-R	CTTTGGTCCTGAGGTGTGCTTC [58]
mKlf4-F	CTATGCAGGCTGTGGCAAAACC	mSox2-F	AACGGCAGCTACAGCATGATGC
mKlf4-R	TTGCGGTAGTGCCTGGTCAGTT [59]	mSox2-R	CGAGCTGGTCATGGAGTTGTAC [60]
mKlf5-F	AGCTCACCTGAGGACTCATACG	mStat3-F	AGGAGTCTAACAACGGCAGCCT
mKlf5-R	AGAAGCTGCGTTGGCACACCAT [59]	mStat3-R	GTGGTACACCTCAGTCTCGAAG [61]
mMafb-F	TACTGGATGGCGAGCAACTACC	mTgfb1-F	TGATACGCCTGAGTGGCTGTCT
mMafb-R	ACTACGGAAGCCGTCGAAGCTC [62]	mTgfb1-R	CACAAGAGCAGTGAGCGCTGAA [63]
mMsx2-F	AAGACGGAGCACCGTGGATACA	mTwist1-F	GATTCAGACCCTCAAACTGGCG
mMsx2-R	CGGTTGGTCTTGTGTTTCCTCAG [53]	mTwist1-R	AGACGGAGAAGGCGTAGCTGAG [64]
mNanog-F	GAACGCCTCATCAATGCCTGCA	mWnt3a-F	AACTGCACCACCGTCAGCAACA
mNanog-R	GAATCAGGGCTGCCTTGAAGAG [60]	mWnt3a-R	AGCGTGTCACTGCGAAAGCTAC [65]
mNes-F	AGGAGAAGCAGGGTCTACAGAG	mWnt5a-F	GGAACGAATCCACGCTAAGGGT
mNes-R	AGTTCTCAGCCTCCAGCAGAGT [66]	mWnt5a-R	AGCACGTCTTGAGGCTACAGGA [67]
mNotch1-F	GCTGCCTCTTTGATGGCTTCGA	mWnt6-F	TTTCCGACGCTGGAACTGCTCC
mNotch1-R	CACATTCGGCACTGTTACAGCC [68]	mWnt6-R	CCTGACAACCACACTGTAGGAG [69]
mNotch2-F	CCACCTGCAATGACTTCATCGG	mWnt10a-F	GCTCCTGTTCTTCCTACTGCTG
mNotch2-R	TCGATGCAGGTGCCTCCATTCT [68]	mWnt10a-R	ATGTCAGGCACACTGTGTTGGC [69]
mOct3/4-F	CAGCAGATCACTCACATCGCCA	mGAPDH-F	GCACAGTCAAGGCCGAGAAT
mOct3/4-R	GCCTCATACTCTTCTCGTTGGG [70]	mGAPDH-R	GCCTTCTCCATGGTGGTGAA [71]
